# Study on identification, assay and organoleptic quality of veterinary medicines in Ethiopia

**DOI:** 10.1186/s40545-022-00410-6

**Published:** 2022-03-03

**Authors:** Belachew Tefera, Belachew Bacha, Sileshi Belew, Ravinetto Raffaella, Tenaw Andualem, Zerihun Abegaz, Ayalew Zelelew, Gudeta Uma, Tadese Setegn, Abdisa Hunduma, Dinsefa Jemal, Diriba Daba, Bizuayehu Belete

**Affiliations:** 1 Veterinary Drug and Feed Administration and Control Authority (VDFACA), Animal Products, Veterinary Drug and Feed Quality Assessment Centre, P.O. Box 31303, Addis Ababa, Ethiopia; 2 Jimma University Laboratory of Drug Quality (JuLaDQ) and School of Pharmacy, Jimma University, P.O. Box 378, Jimma, Ethiopia; 3 Institute of Tropical Medicine, 2000 Antwerp, Belgium; 4 Food and Agriculture Organization of the Untied Nation (FAO), P.O. Box 5536, Addis Ababa, Ethiopia

**Keywords:** Ethiopia, Medicines, Quality, Regulatory authority, Surveillance, Veterinary

## Abstract

**Background:**

Medicines of poor quality are currently prevailing problems undermining the quality of health care services in veterinary and human medicine. In this study, physico-chemical quality of veterinary medicines was evaluated.

**Methods:**

A total of 959 veterinary medicines samples were collected during routine regulatory activities, i.e. pre-registration, re-registration, consignment checking and post-marketing surveillance, in Ethiopia. The samples were transported to Animal Products, Veterinary Drug and Feed Quality Assessment Centre (APVD-FQAC), which is the quality control laboratory of the Veterinary Drug and Feed Administration and Control Authority (VDFACA) and stored until analysis. The samples were subjected to visual inspection and chemical analysis following the United States, European or British Pharmacopoeias, or manufacturer’s methods.

**Results:**

The findings revealed that 12 (1.3%) of tested products showed defects in physical characteristics, packaging, or labelling information, while a total of 66 (6.9%) samples of the investigated products failed to comply with the Pharmacopoeias and supplier’s specification limit set for assay. Of these, 60 samples did not comply with the minimum assay specification limit.

**Conclusion:**

Overall, 8.2% of the investigated veterinary medicine samples did not comply with the specification set for the investigated quality attributes and thus were categorized as of poor quality. This indicates the need for continued strengthening of regulatory functions.

**Supplementary Information:**

The online version contains supplementary material available at 10.1186/s40545-022-00410-6.

## Background

The livestock sector is one of the potential areas that play a critical role in ensuring food security and livelihoods [1, 2]. Thus, there is a shift of farming practices, and an increment and intensification of food animal production [3, 4] which in turn could increase zoonotic and trans-boundary animal diseases that may lead to the increased use of veterinary medicines [5–7]. The global veterinary medicine market expected to reach $85,059.4 million by 2030 [8]. Though veterinary medicines are one of the critical elements in improving productivity of livestock, informal pharmaceutical supply chains and illegal marketing, poor-quality medicines, medicines misuse and inappropriate use are prevailing factors [9–11], that could contribute to treatment failure, antimicrobial resistance development and economic losses [12–15].

In many low- and middle-income countries (LMICs), the National Regulatory Authorities (NRAs) are still under resourced [16]. Even if the World Health Organization (WHO) Global Benchmarking of Regulatory Systems can now be a game changer to guide regulatory strengthening [17], substantial investments will be needed to reach an adequate regulatory maturity [18] everywhere. The weak regulatory systems, scarce resources, poor infrastructure and lack of trained personnel [16, 19–21], contribute to the relatively huge burden of the aforementioned problems in LMICs [20, 22, 23]. Thus, assessing the quality of medicines according to stringent criteria and monitoring their rational use, is crucial, including in the veterinary sector [24] to explore multiplicity and prevalence of the problem and ensure quality health care services.

In Ethiopia, owning to the prevalence of infectious animal diseases [16, 18–20], veterinary medicines such as antibiotics, anthelmintics, antiprotozoals and acaricides are widely used [21, 22]. However, there is scarce information regarding the quality of veterinary medicines circulating in the market [23, 24]. Therefore, the present study was conducted. It was nested in regulatory inspections routinely conducted by the Ethiopian Veterinary Drug and Food Administration and Control Authority (VDFACA). The Authority is a public organization established in 2011 and it is responsible for the registration, licensing, inspection, quality verification and regulation of Veterinary Medicinal Products (VMPs), commercial animal feed and feed supplements. Hence, no VMPs, feed and feed supplements may be produced locally or imported from abroad and put in use, unless it is registered by the Authority after being tested for its quality and safety (proclamation no. 728/2011) [25]. The VDFACA collaborates with the Animal Product, Veterinary Drug and Feed Quality Assessment Center (APVD-FQAC), which is a quality control laboratory established as entity of VDFACA in June 2014. The APVD-FQAC provides laboratory testing services to the public, to verify the quality and safety standards of primary livestock products, VMPs and commercial animal feedstuffs. In this study, the physico-chemical quality of veterinary medicine samples collected by VDFACA during pre-registration, re-registration, consignment check and post-marketing surveillance was assessed at APVD-FQAC.

## Methods

### Study area and design

The study was conducted in Ethiopia which is located in the Horn of Africa. Cross-sectional study was conducted between November 2016 to June 2021 for the purpose of regulatory activities, i.e. pre-registration, re-registration, consignment check and post-marketing surveillance of veterinary medicines.

### Sample collection and sampling techniques

The sample collection of veterinary medicines is routinely conducted by the VDFACA for the purpose of regulatory inspection, i.e. pre-registration, re-registration, consignment check and post-marketing surveillance. We included in the present study, all samples encompassing acaricides, anthelmintics, antibacterials, antiprotozoals and miscellaneous, obtained between 16 November 2016 and 22 June 2021. Samples were provided by the supplier to the VDFACA quality control laboratory when submitting the request for registration or re-registration; or collected during regulatory inspection conducted at import entry check points for consignment or throughout the country for post-marketing surveillance, across different levels of veterinary supply chain. The samples were immediately labelled with a study code and accompanying information of samples was recorded (i.e. sample collection and submission dates, sampling purpose, international nonproprietary name trade name, dosage form, strength, manufacturing and expiry dates, batch number, active pharmaceutical ingredients, storage temperature, stated country of origin, sample collection place, method of analysis). Overall, 959 samples were purposively collected as part of ongoing regulatory inspections.

Samples were properly transported to VDFACA quality control laboratory, Addis Ababa, Ethiopia, for testing, and stored until analysis, according to product manufacturers storage recommendations and in line with WHO Good storage and distribution practices for medical products [26].

### Chemicals, reagents, solvents, and certified reference materials (CRMs)

Methanol, acetonitrile, hydrochloric acid 37% and sulfuric acid 98%, ammonium dihydrogen phosphate, sodium hydroxide, sodium dodecyl sulphate, and sodium dihydrogen orthophosphate were used. All chemicals, reagents and solvents used were analytical grade and met the required purity standards set for specific test and analyses.

### Physico-chemical quality test

All veterinary medicine samples collected for regulatory purpose between 16 November 2016 and 22 June 2021 were subjected to visual inspection by VDFACA experts and to chemical analysis for identity and assay. High-performance liquid chromatography (HPLC-Shimadzu-CTO-20AC) with UV–Vis detector, gas chromatography (GC-Agilent-7890A) with flame ionization detector (FID), UV–Vis spectrophotometer (Jenway-6850) and automatic titrator (Hanon-T860) analytical instruments were used for chemical analyses. The visual inspection encompassing physical characteristics, packaging and labelling information was conducted using the checklist described elsewhere [27] presented in the additional file (see Additional file 1). The chemical analyses were conducted according to the standard methods described in the US, European or British Pharmacopoeias [28] and/or supplied by the manufacturer, in case of in-house specifications.

## Results

### Physico-chemical quality test

The physico-chemical quality of veterinary medicine samples (*n* = 959) encompassing acaricides (*n* = 19), anthelmintics (*n* = 456), antibacterials (*n* = 314), antiprotozoals (*n* = 106), miscellaneous, i.e. vitamins, synchromate hormones, disinfectants, and anaesthesia (*n* = 26) collected during pre-registration, re-registration, consignment check or post-marketing surveillance was evaluated. The descriptions of defects on samples and supplied documents observed during visual inspection are depicted in Table 1. Overall, the results of visual inspection revealed that 12 (1.3%) samples had defects in physical characteristics (Table 2). Out of the total 959 samples examined, 6 (0.6%) of anthelmintic samples submitted for pre-registration were rejected on visual inspection without undertaking any chemical analysis, due to major physical quality defect findings at the time of sample reception.

**Table 1:** Descriptions of defects on the samples and supplied documents observed during visual inspections

Defects	Description of defects
1)Injectable drugs solubility problem	Failure to form homogenous solution upon rigorous shakings (formation of strong coagulation)
2)Failure to meet expected packaging and labelling standards	Labelling information on primary packaging materials such as blisters and sachets manually affixed incorrectly, e.g. manufacturing dates, expiry dates, and batch/lot numbers information and so on
3)The required actual product concentration did not conform to the stated label claim information and quality requirements	Labelling of primary packaging materials describes different concentration and quality attributes of same products
4)Improper labelling information	The labelling information did not match the expected product content. In addition, labelling information was not well readable and visible
5)Defects in test method and validation documents	Incompatible in-house test method and validation document submission for same (a single) product
6)Inspection of certificate of analysis (CoA) that had different assay result specification limits for the same product	Same product, from the same company, having different assay result specification limits, with different Certificate of Analysis (CoA)
7)Nonconformance of vial purity/cleanness	Visible rust formation on vial stopper (cap seal), the rust particle might enter into the injectable solution when later punctured/pierced and hence jeopardize the proper drug administration/treatment practices
8)Mislabelling of VMPs importers (local agents) names and contact addresses	Labelling information on packaging materials did not match drug sample submitter’s (importers/local agent) names and mismatching of contact addresses

**Table 2:** Visual inspection vs. chemical analyses non-compliance summary results (*n* = 959)

S/N	Visual inspection and chemical analyses results	Failed
1	Samples rejected on visual inspections without undergoing any chemical analyses (identification and assay) due to major physical quality defects	0.6% (6/959)
2	Samples subjected to both visual inspections and chemical analyses (identification and assay) and found to be out of specifications for assay	6.9% (66/953)
2.1	Samples that had shown quality defects both in visual inspections and assay result specifications	0.6% (6/953)
2.2	Samples that had shown minor quality defects on visual inspections but passed chemical analyses (identification and assay) specifications and thereby accepted with special corrective measure and preconditions	0.5% (5/953)
2.3	Samples that had no quality defects on visual inspections, but failed to comply with assay result specifications	6.3% (60/953)
3	Total samples did not meet visual inspections and assay result specifications	8.2% (78/959)

The results of identity test indicated that all samples contain the intended active pharmaceutical ingredient (API) that matches with the product’s label claim. However, out of 953 samples investigated for the purpose of regulatory inspection, 66 (6.9%) samples failed to comply with the pharmacopoeia’s and/or suppliers specifications limits regarding the required amount of API contents. Furthermore, 60 samples out of 953 (6.3%) passed the visual inspections, but later failed to comply with assay result specifications.

The assay results of 66 samples deviated from assay specification limit (%label claim (lc.): minimum and maximum) set for each investigated product. The out of specification (OOS) samples assay results are presented in Table 3.

**Table 3:** The OOS samples assay results (%lc.) showing deviation from minimum and maximum assay specification limit

Drug category	Assay specification limit (%)^a^		Number of OOS samples	Number of samples showing OOS assay results deviated from minimum assay limit	Number of samples showing OOS assay results deviated from maximum assay limit
	Min	Max			
Anthelmintic	90	110	38	35	3
Antiprotozoal	95	110	8	8	0
Antibacterial	90	120	15	14	1
Acaricide	90.4	110.4	4	2	2
Miscellaneous^b^	–	–	1	1	0
Total			66	60	6

^a^Indicates assay results specification limit expressed in %lc., ^b^growth of microorganism observed on surgical glove supposed to be sterile

The detailed information on test results (identity and assay) of each sample is presented in the additional file (see Additional file 2).

The results of the quality of medicines categorized based on the source of samples; therapeutic category, level of supply chain and stated production origins are presented in Table 4.

**Table 4:** The results of physico-chemical quality of veterinary medicines samples (*n* = 953)

Category	Samples tested^a^	Pass		Fail	
*Sample source*					
Pre-registration	411	396	96.4%	15	3.6%
Re-registration	98	90	91.8%	8	8.2%
Consignment check	84	80	95.2%	4	4.8%
Post-market survey	360	321	89.2%	39	10.8%
*Supply chain levels*					
Importer	647	618	95.5%	29	4.5%
Wholesaler	30	24	80.0%	6	20.0%
Retailer	276	245	88.8%	31	11.2%
*Therapeutic category*					
Acaricides	19	15	78.9%	4	21.1%
Anthelmintics	488	450	92.2%	38	7.8%
Antibacterials	314	299	95.2%	15	4.8%
Antiprotozoals	106	98	92.5%	8	7.5%
Miscellaneous^b^	26	25	96.2%	1	3.8%
*Stated production origins*					
African countries	47	44	93.6%	3	6.4%
Asia countries	692	639	92.3%	53	7.7%
European countries	176	169	96.0%	7	4.0%
Latin America countries	11	10	9.9%	1	9.1%
Middle East countries	27	25	92.6%	2	7.4%

^a^Indicates number of samples of veterinary medicines categorized under the same or different therapeutic group

^b^Includes vitamins, synchromate hormones, disinfectants, anaesthesia

## Discussion

Quality assured medicines are critical in preventing and mitigating diseases and preventing the emergency of resistance, as well as reducing risks attributed to use of poor-quality medicines. In recent years, there has been growing awareness of the treats to individual and public health represented by poor-quality medicines for human use, but the field of veterinary medicines remains relatively neglected. In this study, nested in routine regulatory activities in Ethiopia, the physico-chemical quality of veterinary medicines was assessed for the purpose of regulatory inspection. The results of this study revealed that 1.3% (12/959) failed the visual inspections, and 6.9% (66/953) of samples failed to comply with the specification limit set for the tested quality attributes. Interestingly, 60 samples out of 953 (6.3%) did not reveal any visible defect when visually inspected, but failed to comply with assay result specifications. This indicates a relatively low predictive value of the visual inspection for this specific determinant of pharmaceutical quality. Though there is scarce information regarding quality of veterinary medicine circulating in Ethiopia, 28% samples of trypanocidal medicines and 50% samples of albendazole brands investigated in previous studies failed to comply with the quality requirements set for the tested quality parameters [29,  30].

The observed poor quality of medicines may reflect failure of pharmaceutical manufacturers to comply with the Good Manufacturing Practice (GMP) [31], or failure to implement adequate storage and distribution practices along the supply chain [26]. In fact, it is difficult to distinguish quality problems caused by poor practices at manufacturing sites vs. those caused by poor practice along the distribution chains. In our study, however, a higher prevalence of out-of-specifications was found during post-market surveillance (10.8%), and at retailers (11.2%) and wholesalers (20%), suggesting the additional impact of poor storage and distribution practices. The lack of stringent medicines regulatory systems, clearly limits countries’ capacity to detect, prevent and respond to poor-quality medicines [32]. This suggests the need for stronger control and monitoring of quality of veterinary pharmaceuticals during production, procurement, distribution/supply, storage, and surveillance. It is hoped that the forthcoming African Medicine Agency (AMA) [33] will represent a game changer for strengthening regulatory oversight across the continent, including for veterinary medicines.

It is well known that administering poor-quality medicines (i.e. substandard and falsified) [34] leads to poor or reduced treatment outcomes, toxicity and antimicrobial resistance [35–37], in addition to causing an unnecessary economic burden on the health system [38]. The poor quality of about 8.2% of the medicines investigated in this study implies a potential negative consequence also in the animal disease prevention and control efforts, as well as economy of the country. In areas where there is application of inappropriate dosage and incorrect duration of veterinary medicines due to lack of knowledge and involvement of non-professionals [39–42], the impact might be even higher. In Ethiopia, weak medicine regulation and involvement of non-professional actors in the veterinary sector [43, 44] could contribute to the infiltration of poor-quality medicines into medicines supply chain. Furthermore, all medicines analysed in this study were subjected to regulatory oversight; it may be hypothesized that the prevalence of out-of-specifications would be higher for medicines circulating in the informal market. Given that fact that veterinary medicines such as tetracyclines, albendazole and diminazene diaceturate are essential medicines widely used in veterinary sectors in Ethiopia, the quality failure observed for these medicines could jeopardize the existing efforts in veterinary services [44, 45].

Our study has a few limitations: for instance, we could not make distinctions between products originally of poor quality and those that degraded along the supply chain; we only sampled product available in the formal sector; and we did not carry out all the Pharmacopoeial tests, e.g. we did not conduct dissolution and impurity tests. Also, not all the regions in Ethiopia were equally represented. Thus, the findings of this report are likely to be the iceberg of the real situation on the ground and points to the need for further actions in conducting wide-ranging studies. Such studies should either be nested in regulatory activities, or conducted in partnership with the regulatory authorities, so as to provide them with immediate guidance on prevalence and distribution of poor medicines, for corrective measures.

## Conclusions

The results of the present study indicated that 8.2% the investigated samples of veterinary medicines failed to comply with one or more of the assessed quality standards. Therefore, continued strengthening of the inspection of veterinary medicines and drug regulatory functions is highly recommended.

## Supplementary Information

**Additional file 1:** Checklist.

**Additional file 2:** Identity and assay results.

## Data Availability

All the data and materials will be made available on request to the corresponding author.

## Abbreviations

AMA: African Medicine Agency
APVD-FQAC: Animal Products, Veterinary Drug and Feed Quality Assessment Centre
BP: British Pharmacopoeia
CoA: Certificate of Analysis
CRMs: Certified Reference Materials
EDQM: European Directorate for the Quality of Medicine
EP: European Pharmacopoeia
FAO: Food and Agriculture Organization of the Untied Nation
GC: Gas chromatography
GMP: Good Manufacturing Practice
HPLC: High-performance liquid chromatography
JuLaDQ: Jimma University Laboratory of Drug Quality
LMICs: Low- and middle-income countries
NRAs: National Regulatory Authorities
USP: US Pharmacopoeia
