# Correction to: Study on identification, assay and organoleptic quality of veterinary medicines in Ethiopia

**DOI:** 10.1186/s40545-022-00437-9

**Published:** 2022-06-08

**Authors:** Belachew Tefera, Belachew Bacha, Sileshi Belew, Rafaella Ravinetto, Tenaw Andualem, Zerihun Abegaz, Ayalew Zelelew, Gudeta Uma, Tadese Setegn, Abdisa Hunduma, Dinsefa Jemal, Diriba Daba, Bizuayehu Belete

**Affiliations:** 1 Veterinary Drug and Feed Administration and Control Authority (VDFACA), Animal Products, Veterinary Drug and Feed Quality Assessment Centre, P.O. Box 31303, Addis Ababa, Ethiopia; 2 Jimma University Laboratory of Drug Quality (JuLaDQ), School of Pharmacy, Jimma University, P.O. Box 378, Jimma, Ethiopia; 3 Institute of Tropical Medicine, 2000 Antwerp, Belgium; 4 Food and Agriculture Organization of the Untied Nation (FAO), P.O. Box 5536, Addis Ababa, Ethiopia

## Correction to: Journal of Pharmaceutical Policy and Practice (2022) 15:17 https://doi.org/10.1186/s40545-022-00410-6

Following the publication of the original article [1], we were notified that the first and last name of the 4th author are swapped. Ravinetto Raffaella should be Raffaella Ravinetto.

The original article has been corrected.

## References

[1] Tefera B, Bacha B, Belew S, Ravinetto R, Andualem T, Abegaz Z, Zelelew A, Uma G, Setegn T, Hunduma A, Jemal D, Daba D, Belete B (2022). Study on identification, assay and organoleptic quality of veterinary medicines in Ethiopia. J of Pharm Policy and Pract.

