# Supplementary material for: Study on identification, assay and organoleptic quality of veterinary medicines in Ethiopia
Source: J Pharm Policy Pract. 2022 Mar 3;15:17. doi: 10.1186/s40545-022-00410-6 (PMC8892721; doi:10.1186/s40545-022-00410-6)
Supplement: Supplementary file 1 — Additional file 1: Checklist. [file 40545_2022_410_MOESM1_ESM.docx]

**Supplementary information**

**S1 Table**

Physical characteristics, packaging and labelling information of samples.

| # | Brand/Generic | Packaging | | | | | | | | | | | | | Physical characteristics (visual) | | | | | | | |
| --- | --- | --- | --- | --- | --- | --- | --- | --- | --- | --- | --- | --- | --- | --- | --- | --- | --- | --- | --- | --- | --- | --- |
|  |  | Container and closure (yes/no) | Label completeness (yes/no) | | | | | | | | | | | Leaflet or package insert | Uniformity of shape | Uniformity of size | Uniformity of colour | Uniformity of texture | Markings | Breaks, Cracks and Splits | Embedded surface spots or contamination | Smell |
|  |  |  | Trade/brand name | Active ingredient name | Manufacturer's name and logo | Manufacturer's full address | Medicine strength (mg/unit) | Dosage form | No. of units per container | Dosage statement | Batch/lot No. | Manufactury and expiry date | Storage information |  |  |  |  |  |  |  |  |  |
|  |  |  |  |  |  |  |  |  |  |  |  |  |  |  |  |  |  |  |  |  |  |  |
|  |  |  |  |  |  |  |  |  |  |  |  |  |  |  |  |  |  |  |  |  |  |  |
|  |  |  |  |  |  |  |  |  |  |  |  |  |  |  |  |  |  |  |  |  |  |  |
